# Intraoperative parathyroid hormone assay during focused parathyroidectomy: the importance of 20 minutes measurement

**DOI:** 10.1186/1471-2482-13-36

**Published:** 2013-09-18

**Authors:** Pietro Giorgio Calò, Giuseppe Pisano, Giulia Loi, Fabio Medas, Lucia Barca, Matteo Atzeni, Angelo Nicolosi

**Affiliations:** 1 Department of Surgical Sciences, University of Cagliari, Cagliari, Italy; 2 School of Specialty in Clinical Pathology, University of Cagliari, Cagliari, Italy

**Keywords:** Primary hyperparathyroidism, Parathyroid hormone, Parathyroidectomy, Intra-operative PTH

## Abstract

**Background:**

Parathyroid hormone (PTH) monitoring during the surgical procedure can confirm the removal of all hyperfunctioning parathyroid tissue, as the half-life of PTH is approximately 5 min. The commonly applied Irvin criterion is reported to correctly predict post-operative calcium levels in 96-98% of patients. However, the PTH baseline reference concentration is markedly influenced by surgical manipulations during preparation of the affected glands, interindividual variability of the PTH half-life and modifications in the physiological state of the patient during surgery. The aim of this study was to evaluate the possible impact of the measurement of intraoperative PTH 20 minutes after surgery.

**Methods:**

Between 2003 and 2012, 188 patients underwent a focused parathyroidectomy associated to rapid intraoperative PTH assay monitoring. Blood samples were collected: 1) at pre-incision time, 2) at 10 min after gland excision and 3) at 20 min after excision, if a sufficient reduction of PTH value was not observed. On the bases of the Irvin criterion, an intra-operative PTH drop>50% from the highest either pre-incision or pre-excision level after parathyroid excision was considered a surgical success.

**Results:**

A >50% decrease of PTH after gland excision compared to the highest pre-excision value occurred in 156/188 patients (83%) within 10 min and in further 12/188 after 20 minutes (6.4%). In the remaining 20 patients (10.6%) values of PTH remained substantially unchanged or decreased less than 50% and for this reason bilateral neck exploration was performed. An additional pathologic parathyroid was removed in 9 cases, a third in one. In the other 10 cases further neck exploration by a standard cervical approach was negative and in four of these persistent postoperative hypercalcemia was demonstrated. The overall operative success was 97.3%. Intraoperative PTH monitoring was accurate in predicting operative success or failure in 96.3% of patients.

**Conclusions:**

The 20 minutes PTH measurement appears very useful, avoiding unnecessary bilateral exploration and the related risk of complications with only a slight increase of the duration of surgery and of the costs. PTH values decreasing appeared to be influenced by surgical manipulations during minimally invasive parathyroidectomy.

## Background

Primary hyperparathyroidism is a common condition caused by single or multiple parathyroid lesions [1,2]; it is rare below the age of 50 years but rises thereafter, particularly in women; surgery offers the only definitive treatment [2].

The aim of parathyroidectomy is to establish normocalcaemia trying to avoid complications such as persistent or recurrent hyperparathyroidism, postoperative transient or persistent hypoparathyroidism and recurrent laryngeal nerve injury [2].

Primary hyperparathyroidism has traditionally been managed by bilateral neck exploration and identification of the four parathyroid glands with a success rate of more than 95% when performed by experienced endocrine surgeons [2-6].

The traditional surgical approach with the visualization of all parathyroid glands and the resection of apparently enlarged glands has been increasingly replaced by minimally invasive (unilateral) surgical procedures, supported by preoperative imaging and rapid intraoperative parathyroid hormone (PTH) assay measurement [4,6-8].

However, 5% to 20% of patients with primary hyperparathyroidism have multiglandular disease and require bilateral neck exploration; in such cases, imaging studies can be misleading [6].

PTH monitoring during the surgical procedure can confirm the removal of all hyperfunctioning parathyroid tissue, as the half-life of PTH is approximately 5 min [2-4,7]. An insufficient decrease in PTH indicates persisting primary hyperparathyroidism, leading to more extended (bilateral) exploration within the same session [3,4,7].

The commonly applied Irvin criterion (rapid intra-operative PTH assay drop ≥ 50% from the highest value of either pre-incision or pre-excision level at 10 min after gland excision) is reported to correctly predict post-operative calcium levels in 96-98% of patients and incorrectly in only 2-4% [3,6,7,9]. Some authors suggest to fulfill various percentage drop versus pre-incision or pre-excision PTH, 5-10 min after resection of the suspected parathyroid adenoma, or to reach a final PTH concentration within the normal range [3,4].

However, the PTH baseline reference concentration is markedly influenced by surgical manipulations during preparation of the affected glands, interindividual variability of the PTH half-life and modifications in the physiological state of the patient during surgery (such as changes of clearing rate or expanded volume of intra-operative infusions) [4,7,10].

Some authors have reported error rates of as much as 16% due to false-negative and false-positive results [3,11].

The aim of this study was to evaluate the role of the measurement of intraoperative PTH 20 minutes after surgery.

## Methods

We conducted a retrospective study on 239 patients operated on for primary hyperparathyroidism in our surgical department between May 2003 and December 2012. 202 patients were female and 37 were male, median age was 58 years (range 19-85). Before operation hypercalcemia and elevated PTH levels were observed in all patients.

In order to localize hyperfunctioning glands, a TC99m-sestamibi scan (MIBI) was performed in 191 patients (79.9%): pathologic parathyroid was localized in 178 cases (93.2%). High resolution ultrasound was associated in 233 patients and pathologic parathyroid was localized in 146 (62.7%). Association of ultrasound and 99m Tc-sestamibi scan localized hyperfunctioning parathyroid in 163/174 patients (93.7%). SPECT-TC was performed on 140 patients and hyperfunctioning parathyroid was localized in 134 (95.7%). Association of SPECT-TC and ultrasonography localized hyperfunctioning parathyroid in 121/122 patients (99.2%).

188 patients underwent a focused parathyroidectomy associated to a rapid intraoperative PTH assay monitoring. All patients had normal renal function (serum creatinine value ranging from 0.7 to 1.2 mg/dL) and gave informed consent for the procedure. All operations were performed under general anesthesia with endotracheal intubation and by the same team of surgeons, who were highly experienced in parathyroid surgery. Blood samples were collected: 1) at pre-incision time, 2) at 10 min after gland excision and 3) at 20 min after excision, if a sufficient reduction of PTH value was not observed. The STAT-IntraOperative-Intact-PTH Chemilluminescence immunoassay semiautomated mobile system (Future Diagnostics, Wijchen, Netherlands) was used within the surgical suite complex for the intraoperative quantitative determination of PTH levels in EDTA plasma.

The study has been performed in accordance with the Declaration of Helsinki. Ethical approval for our study was obtained from institutional ethical committee of Monserrato University Hospital, Cagliari, Italy. Informed consent was obtained from participants for their inclusion in our study.

On the bases of the Irvin criterion, an intra-operative PTH drop >50% from the highest either pre-incision or pre-excision level after parathyroid excision was considered a surgical success. A PTH drop of <50% from the highest basal value within 20 min after gland excision and/or a residual PTH-20 min level higher than the reference range were considered predictor of persistent hyperfunctioning parathyroid tissue and further surgical exploration was required.

## Results

A >50% decrease of PTH within 10 min compared to the highest pre-excision value occurred in 156/188 patients (83%). In 12 patients >50% decrease of PTH was obtained after 20 minutes (6.4%) (Table 1). The characteristics of the 12 patients of this group are shown in Table 2. Total >50% decrease of PTH within 20 minutes was obtained in 168/188 patients (89.4%); postoperative normal calcemia and PTH were found in 167 patients and persistent postoperative hypercalcemia with increased value of PTH was found in 1 patient. In the remaining 20 patients (10.6%) values of PTH remained substantially unchanged or decreased less than 50%. In these patients the surgical procedure went on and bilateral neck exploration was performed. An additional pathologic parathyroid was removed in 9 cases, a third in one case. In these cases histology exams showed hyperplasia in 5 cases, double adenoma in 4 and single adenoma associated to hyperplasia in 1. In the other 10 cases further neck exploration by a standard cervical approach was negative and in four of these persistent postoperative hypercalcemia was demonstrated, whereas in six patients postoperative normal calcemia and PTH were found.

**Table 1:** Decrease of PTH

**Decrease of PTH**	**Patients**	**%**
>50% decrease of PTH within 10 min	156/188	83%
>50% decrease of PTH after 20 min	12/188	6.4%
No >50% decrease of PTH	20/188	10.6%

**Table 2:** Characteristics of the 12 patients in which >50% decrease of PTH was obtained after 20 minutes

	
Mean age	58.25
Positive and concordant localization studies	9/12
Negative or discordant localization studies	3/12
Mean baseline PTH (pg/ml)	170.33

Final histology showed a parathyroid adenoma in 150 patients, hyperplasia in 35, carcinoma in 3.

The overall operative success was 97.3%, with a 4.8% incidence of multiglandular disease (9 cases).

Median duration of surgery was 60 minutes (range 40-80). Surgical complications have been observed in 3 patients: 2 transient recurrent laryngeal nerve paresis and 1 hematoma necessitating reoperation. Transient hypocalcemia occurred in 24 cases (12.7%) and was treated with calcium and vitamin D per os with resolution within 1 month. No cases of permanent hypocalcemia were observed. There were no wound complications and no perioperative deaths.

Median postoperative recovery was 2 days (range 1-3). Intraoperative PTH monitoring was accurate in predicting operative success or failure in 96.3% of cases. Thus the method had a sensitivity of 96.5%, a specificity of 93.3%, a positive predictive value of 99.4% and a negative predictive value of 70% (Table 3).

**Table 3:** Results of intraoperative PTH monitoring in focused parathyroidectomy

	
Sensitivity	96.5%
Specificity	93.3%
Positive predictive value	99.4%
Negative predictive value	70%
Overall accuracy	96.3%

We compared the characteristics of patients in which there was a >50% decrease of PTH within 10 minutes (group A) to those with no decrease or late decrease (group B). We found no significant difference for the age and for preoperative localization studies, while the mean value of baseline PTH was significantly lower in the first group (Table 4).

**Table 4:** Comparison between patients in which there was a >50% decrease of PTH within 10 min (group A) to those with no decrease or late decrease (group B)

	**Group A**	**Group B**	**p**
Patients	156	32	
Mean age	59.4	59.9	0.84
Positive and concordant localization studies	106	18	0.2857
Negative or discordant localization studies	50	14	
Mean baseline PTH (pg/ml)	334.81	152.67	0.004

## Discussion

Currently, the success rate of parathyroidectomy is greater than 95% in centers experienced in this procedure and complications are rare [2,12,13].

Intraoperative PTH monitoring is a necessary adjunct to detect multiglandular disease in patients undergoing minimally invasive surgery for primary hyperparathyroidism [14]. Pitfalls may occur due to technical assay problems or blood sampling errors [10]. In particular, blood collection timing appears to be a critical step [4].

Difficulties or unexpected outcomes can be encountered in patients with renal insufficiency and when PTH spikes with consecutive delayed PTH reduction occur by unintended manipulation of parathyroid adenomas during mobilization [10]. In particular, during minimally invasive parathyroidectomy, the manipulations by the surgeons influence the PTH concentrations values until parathyroidectomy obviously to a larger extent than with conventional techniques. The preparation of the affected gland within a small unilateral 1.5-cm skin incision may cause squeezing of the glands as a result of mobilization or untimely clamping of small vessels. Extirpation of the adenoma no doubt stopped its PTH efflux into circulation, irrespective of a previous stepwise clamping [7]. If unintended manipulation results in a PTH peak, the PTH decay is not exponential, and the criterion of 50% reduction of the pre-incision baseline level is delayed to more than 10 min in most patients after extensive manipulation. The PTH decay after manipulation may be even prolonged in patients with impaired renal function [10,15,16].

A slow decrease in PTH may also be caused by a prolonged half-life and not necessarily by additional hyperfunctioning glands [7].

Prolonged PTH monitoring at 20 min was suggested by some authors which found that the best predictors of operative failure and persistent (multiglandular) disease are a PTH drop of <50% at 20 min and/or a residual (at 20 min) PTH level above the normal range and/or a significant increase of the PTH levels between the sample obtained 20 min after gland excision with the respect to the 10-min samples [3,4,17,18].

We performed PTH measurement after 10 minutes and if a sufficient decrease of PTH value was not observed after 20 minutes: only after this second measurement we perform bilateral exploration. In 12 patients (6.4%) >50% decrease of PTH was observed only after 20 minutes and an unnecessary bilateral exploration was thus avoided. None of the 12 patients had impaired renal function nor other comorbidities, so the delayed normalization of PTH appears to be probably caused by the excessive manipulation during the minimally invasive parathyroidectomy.

In 6 patients, in which a >50% decrease of PTH in the 20 min sample was not obtained, postoperative normal calcemia and PTH were found. In these cases, it is possible that a further sample after 30 minutes could have obtained a >50% decrease. Possible explanations for these unexpected outcomes are technical assay problems or blood sampling errors; in particular unintended manipulation with a PTH spike could have resulted in a delay of more than 20 minutes.

Baseline PTH values in patients without decrease or with late decrease of PTH were significantly smaller than those with a >50% decrease of PTH within 10 min. The reason for this is not clear: it could be attributable to PTH peaks due to manipulations that can obviously have a more significant effect in patients with lower baseline PTH values; moreover, lower PTH values generally correspond to smaller parathyroid and more difficulties in their intraoperative localization.

Our results are similar to those reported in literature [4-6,17] showing an accuracy in predicting operative success or failure in 96.3% of cases, a sensitivity of 96.5%, a specificity of 93.3%, a positive predictive value of 99.4% and a negative predictive value of 70%. Our incidence of multiglandular diseases (4.8%) was instead only slightly lower than that reported in literature (5-20%) [3,6,8,19], and in these cases a correct diagnosis and a proper treatment have been obtained.

## Conclusions

Our approach with the PTH 20 minutes measurement when the value does not decrease significantly at 10 minutes made it possible to achieve a significant accuracy, sensitivity and specificity of the method avoiding unnecessary bilateral explorations and associated complications in patients undergoing minimally invasive surgery. Duration of surgery and related costs were only minimally increased.

Monitoring of the PTH decay appeared to be influenced by surgical manipulations during the more difficult adenoma preparation in minimally invasive parathyroidectomy.

The 20 minutes end-point seems to play a key role in the correct determination of surgical outcome, strongly improving the possibility of performing correct patient treatment.

So the 20 minutes PTH measurement appears very useful, avoiding unnecessary bilateral exploration and the related risk of complications with only a slight increase of the duration of surgery and its costs.

## Competing interests

The authors declare that they have no competing interests.

## Authors’ contributions

PGC carried out part of the operations, conceived of the study and drafted the manuscript. GP carried out part of the operations and revised the article. GL participated in the design of the study and in the acquisition of the data. FM participated in the design of the study and performed the statistical analysis. LB carried out all the PTH measurements and participated in the acquisition of the data. MA revised the article and participated in the acquisition of the data. AN participated in the design of the study and helped to draft the manuscript. All authors read and approved the final manuscript.

## Pre-publication history

The pre-publication history for this paper can be accessed here:

http://www.biomedcentral.com/1471-2482/13/36/prepub
